# Bladder Perforation due to Hospital Acquired Delirium

**DOI:** 10.51894/001c.144540

**Published:** 2025-09-30

**Authors:** Mercedes Kent

**Affiliations:** 1 Department of Urology University of Michigan Health Sparrow

## 93

### INTRODUCTION

Artificial urinary sphincters (AUS) provide a surgical option for addressing stress urinary incontinence (SUI) in men. SUI is a common complication after radical prostatectomy performed by prostate cancer and AUS are most commonly used in this setting. The device requires manual dexterity from the patient in order to properly deactivate the device therefore deflating the artificial sphincter and allowing urination. In the appropriate patient population these can be highly successful with excellent patient satisfaction.

### CASE DESCRIPTION

JF is an 87 year old male with a urologic history notable for prostate cancer who underwent radical prostatectomy and radiation therapy (2008) followed by placement of AUS (2013) for associated stress incontinence. In 02/2024 he was admitted for viral respiratory infection later complicated by hospital acquired delirium. Due to change in mentation, the AUS was unable to deactivated for urination and therefore urinary retention, ultimately bladder perforation. Urology was consulted after concerning physical exam and CT A/P demonstrating possible perforation.

The primary service was not aware of his AUS rather believed he had a penile prosthesis. Therefore his sphincter had remained activated since the episode of delirium began approximately 24 hours prior to time of consult. CT cystogram confirmed the injury. The patient was taken for emergent surgery to repair the injury via robotic assisted cystorrhaphy. Post-operatively he required ICU management.

### DISCUSSION/CONCLUSION

In the ideal patient population, artificial urinary sphincters provide an excellent option for males to maintain continence and therefore quality of life. However this case study demonstrates the importance monitoring urinary output and mental status in those patients who are hospitalized. Hospital acquired delirium is not uncommon the elderly population and therefore it stresses the importance of communication between primary and urologic services in order to deactivate devices when needed to avoid this serious complication.

